# Evidence-Based African Swine Fever Policies: Do We Address Virus and Host Adequately?

**DOI:** 10.3389/fvets.2021.637487

**Published:** 2021-03-24

**Authors:** Frank Busch, Céline Haumont, Mary-Louise Penrith, Alberto Laddomada, Klaas Dietze, Anja Globig, Vittorio Guberti, Laura Zani, Klaus Depner

**Affiliations:** ^1^Institute of International Animal Health/One Health, Friedrich-Loeffler-Institute, Greifswald, Germany; ^2^National College of Veterinary Medicine, Food Science and Engineering, Oniris, Nantes, France; ^3^Department of Veterinary Tropical Diseases, Faculty of Veterinary Science, University of Pretoria, Pretoria, South Africa; ^4^Consultant, Cagliari, Italy; ^5^Istituto Superiore per la Protezione e la Ricerca Ambientale, Epidemiology and Ecology Unit, Ozzano Emilia, Italy

**Keywords:** African swine fever, ASF policies, ASF surveillance, disease control, legislation, backyard farm, transboundary animal disease, contagiousness

## Abstract

African swine fever (ASF) is one of the most threatening diseases for the pig farming sector worldwide. Prevention, control and eradication remain a challenge, especially in the absence of an effective vaccine or cure and despite the relatively low contagiousness of this pathogen in contrast to Classical Swine Fever or Foot and Mouth disease, for example. Usually lethal in pigs and wild boar, this viral transboundary animal disease has the potential to significantly disrupt global trade and threaten food security. This paper outlines the importance of a disease-specific legal framework, based on the latest scientific evidence in order to improve ASF control. It compares the legal basis for ASF control in a number of pig-producing regions globally, considering diverse production systems, taking into account current scientific evidence in relation to ASF spread and control. We argue that blanket policies that do not take into account disease-relevant characteristics of a biological agent, nor the specifics under which the host species are kept, can hamper disease control efforts and may prove disproportionate.

## Introduction

Like other transboundary animal diseases (TADs), African swine fever (ASF) can impact economies in affected countries significantly due to losses in trade, pig production and associated food security threats (1, 2). Whilst ASF virus (ASFV) continues to spread among domestic pigs (*Sus scrofa domesticus*) and wild boar (*Sus scrofa*) in large areas of Eurasia, many aspects regarding the key mechanisms that drive infection transmission and disease persistence are yet to be fully understood (3, 4). Legal frameworks that underpin animal health interventions must take into consideration the biology of an infectious agent as well as the host species and, if domestic, the production systems, in order to develop appropriate and targeted strategies to combat the disease.

Whilst it is commonly accepted that ASF disease control in wild boar warrants a tailored approach, no special dispensation exists for domestic pigs, despite the fact that differences in the epidemiology of ASF have been observed in the various production systems: e.g., commercial industrial farming vs. traditional pig farming systems with backyard and smallholders or even free-ranging, feral pigs (5–7).

Where evidence emerges, based on scientific studies and/or well-documented field observations, that aspects of current strategies could be improved, efforts must be made to amend the relevant animal health legislation accordingly in order to ensure a progressive and measured disease control approach.

The aim of this paper is (i) to compare ASF-related legislation and the prescribed disease control and eradication measures for domestic pigs from countries spanning five continents (Africa, Asia, Europe, America, Oceania) and covering over 75% of the global pig population, and (ii) to analyze their applicability, taking into account our current understanding of the disease, drawing from global ASF experiences.

The authors are discussing disease control policies in relation to highly virulent ASF strains as the genotype II virus that is currently circulating in Eurasia.

## Disease Control and Eradication Measures of ASF Across the Globe

**Figure 1:**
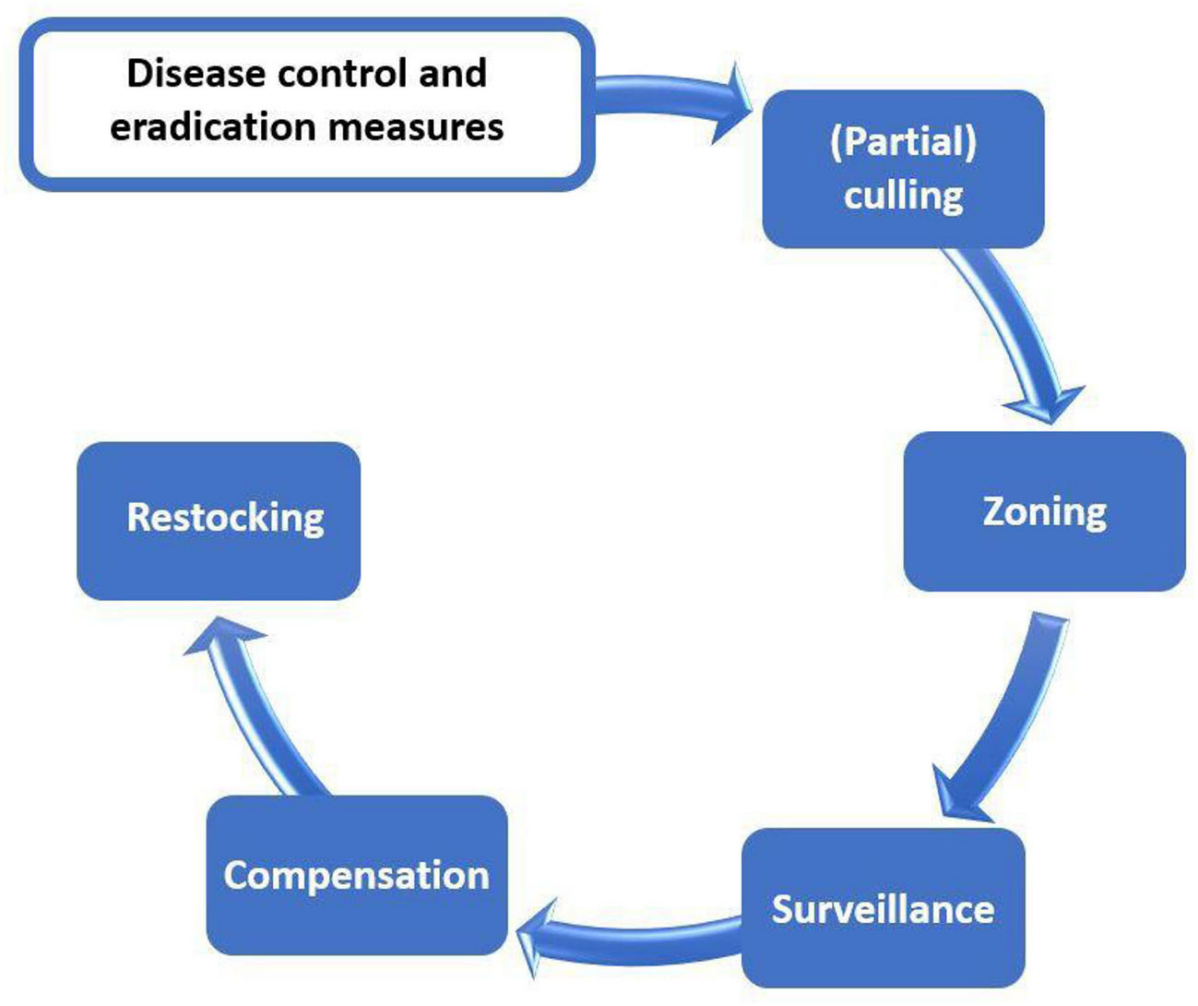
Schematic view of ASF control and eradication measures across the globe.

The control of ASF in domestic pigs follows the general concepts recommended for controlling transboundary animal diseases: As soon as the presence of the disease is suspected, a number of specific diagnostic actions must follow in order to confirm or exclude the presence of disease. Once ASF is confirmed, the infected holding must be isolated and depopulated, partially or entirely, although this is not always possible due to socio-economic constraints, for example. Further spread must be prevented through immediate cessation of animal movements (standstill), the tracing of contact holdings and potentially contaminated products, and through the establishment of surveillance and protection zones around the index case. The aim of these activities is to eradicate the disease within the affected area, prevent the spread outside of it and at the same time allow trade and movements of animals and animal products outside the restricted areas in order to minimize disruption to the pig value chain (Figure 1).

European legislation aims to harmonize measures for Member States regarding disease control and eradication and the measures therein (Figure 1) are very prescriptive. The measures laid down in the current European Union (EU) legislation represent the minimum set of measures that must be implemented.

Outside of the EU, ASF-related legislation seems less prescriptive. In the case of the United States of America (USA), instructions are limited and guidance is provided based on a number of disease scenarios and on a case-by-case basis. The relevant documents advise that it is more effective to share distinct, concise, flexible policy guidance, as an outbreak unfolds, in order to adapt it rapidly to a specific situation. Therefore, measures and protocols differ between USA-States (8). The United States Department of Agriculture (USDA) stresses the use of strategies that (a) detect, control, and contain the disease in animals as quickly as possible, (b) eradicate the disease using strategies that seek to stabilize animal agriculture, the food supply, the economy, and to protect public health and the environment and (c) provide science- and risk-based approaches and systems to facilitate continuity of business for non-infected animals and non-contaminated animal products (8). In contrast to the 2013 USDA ASF response plan, the updated response plan (2020) has further developed different components of control and eradication (including feral pig management, culling guidelines and others) resulting in a more comprehensive guidance aiming to harmonize procedures between USA-States. For example, well-defined radii for zoning are now provided, which constitutes a common and solid reference to rely on before adjusting them to the epidemiological situation of a given outbreak (8).

Australian ASF-legislation focuses particularly on reassessments of decisions taken following unfolding epidemiological events. The legislation envisages the possibility of a transition to a long-term control policy if eradication is deemed to be impossible (9).

Chinese and Russian ASF legislation employ stamping out policies without exceptions; case- by-case approaches are not permissible (10, 11).

Vietnamese ASF-legislation appears to provide some flexibility, stating that “provinces and cities develop plans for pig production areas appropriate to local practical conditions” (12). It also expresses the need to cooperate with international, intergovernmental and non-governmental organizations and mentions, besides others, the Food and Agriculture Organization (FAO), the World Organization for Animal Health (OIE), and the Association of Southeast Asian Nations (ASEAN); it suggests to seek out cooperation with neighboring countries, in particular China, as the geographically closest ASF-infected country, in order to obtain regional information and benefit from technical and financial assistance (12).

Although total eradication of ASF is not possible in South Africa due to natural vectors and hosts, the disease can be successfully controlled and eradicated in domestic pig production systems if contact with the virus is eliminated (13–15). The strategy is that of long-term control with an emphasis on prevention. Three types of pig farms are permitted in the ASF controlled areas where the disease is endemic in the warthog-tick sylvatic cycle, namely compartments, accredited and listed piggeries as defined by law (13). Compartments comply with international standards provided in Chapters 4.4 and 4.5 of the Terrestrial Animal Health Code of the OIE and South African legislation for pig compartments (16). Compartments have the highest level of biosecurity and may supply the export market regardless of the status of the area where they are situated (17). Accredited farms are registered farms that comply with biosecurity standards laid down in the abovementioned legislation for control of ASF and may supply pigs for slaughter outside the control area but only to non-export abattoirs. Listed farms are those that are registered and maintain basic biosecurity measures but may only supply pigs for slaughter to abattoirs within the control area (13). The South African legislation allows for flexibility since action plans for investigation and control must be developed by the respective farmer/owner of the pig herd in consultation with the local State Veterinarian (13). The strategy cited is an initiative to improve this situation.

Summarizing coordinated efforts across the African continent, however, the FAO, the African Union—Interafrican Bureau for Animal Resources (AU-IBAR) and the International Livestock Research Institute (ILRI) observe that “there is lack of intra-regional cooperation toward the control of the disease in Africa” (18).

The variations in the disease control and eradication measures of the countries and regions included in this paper are summarized in Tables 1A, B.

Table 1A selection of disease control and eradication measures in the international context.

**Table:** 

**Measures**	**EU**	**USA**	**South Africa**	**Russia**	**Australia**
**A**					
Stamping out	Mandatory	Mandatory	No	Mandatory	Mandatory
	All pigs in the infected holding	All pigs in the infected holding	Quarantine preferred	All pigs in the 1st zone	All pigs in the infected and highly suspected holdings
Zoning	Yes 3 km 10 km	Yes 3 km 2 km buffer zone 10 km	No but permanent “controlled areas” for endemic ones	Yes 5–20 km 100–150 km	Yes 3 km 10 km
Standstill of animal movements	Yes In restricted zones	Yes	Yes In the infected property	Yes	Yes In restricted zones
Surveillance	Active and passive	Active and passive	Yes if resources (active, passive)	Yes Type (active or passive) unclear	Active and passive
Compensation	Up to 100%	50% of market value	No	Not specified	50% government 50% industry
Lifting of restrictions	Min. 30 days after C and D	Min. 30 days after C and D	3 months after the last case	6 months after end of quarantine	Min. 30 days after C and D
Restocking	Min. 40 days after C and D	Variable	Not specified	One year after end of quarantine	Min. 6 weeks after C and D
Sentinel animals	Variable	Variable	Variable	No	Yes
Frequency of legislation's review	No mention	“As needed”	No mention	No mention	“As needed”
	Last update in 2002	Last update in 2020	Last update in 2018	Last update in 1980	Last update in 2016

**Table:** 

**Measures**	**Canada**	**China**	**Vietnam**	**Japan**
**B**				
Stamping out	Mandatory	Mandatory	Variable	Mandatory
	All pigs on any site where testing indicates ASF-presence	All pigs in the infected holding	Only pigs with (+) test results	All pigs in the infected holding
Zoning	Yes 1st zone: no radius specified 2nd zone: 10 km	Yes 3 km 10 km 50 km if wild boar activity	Yes 3 km 10 km	Yes 3 km 10 km
Standstill of animal movements	Yes	Yes	Variable Not for pigs tested (-)	Yes
Surveillance	Passive	Active and passive	Active	Yes
				Type (active or passive) not specified
Compensation	Yes Up to 5,000 Can.$/culled pig	Variable *Pro rata* basis	38,000 VND/kg pig (1,49€/kg)	100%
Lifting of restrictions	3 months after C and D	21 days after C and D	2 months after C and D	22 days after C and D
Restocking	If sentinel pigs are (-) after 2 months	If sentinel pigs are (-) after 45 days	30 days after the last case	Min. 6 weeks after C and D
		Or if environment is (-) after 5 months empty		
Sentinel animals	Yes	Optional	Yes	Yes
Frequency of legislation's review	“As needed”	No mention	“As needed”	Every 3 years
	Last update in 2019	Last update in 2020	Last update in 2020	Last update in 2019

*C, Cleaning; D, Disinfection*.

## Legislation and Contagiousness

When ASF-legislation was formulated for the EU, ASF was defined to be a highly contagious disease and such references still prevail (19). However, analyses of domestic pig outbreaks in the current epizootic in Europe, as well as in experimental studies, revealed that the contagiousness of ASF is comparatively low and that under field conditions ASFV transmission between animals is considered to be slow (20, 21).

**Figure 2:**
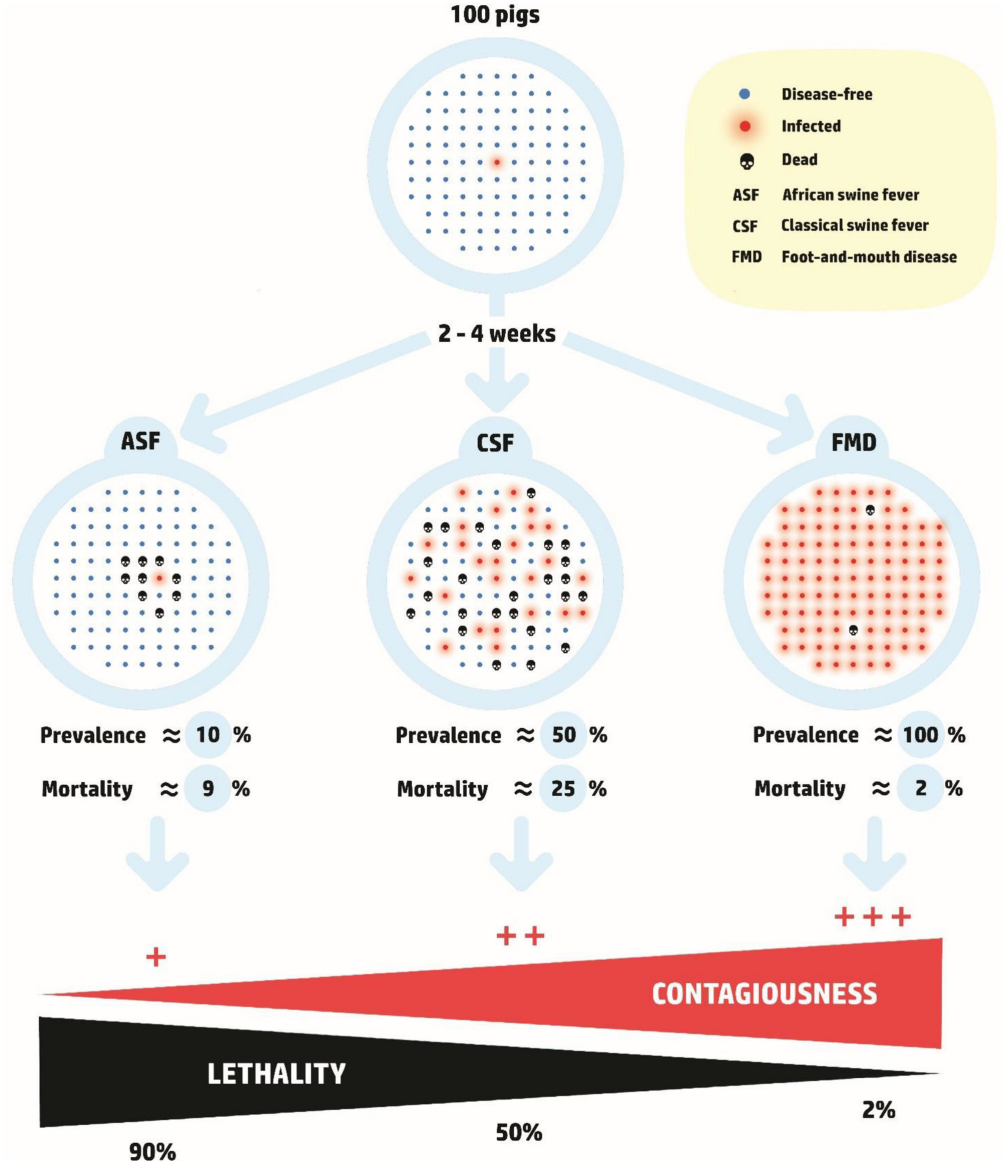
Hypothetical disease scenario of three major pig diseases over a period of 2–4 weeks (22), highlighting the differences between Foot and Mouth Disease (FMD), Classical Swine Fever (CSF) and African Swine Fever (ASF). In comparison to FMD and CSF, contagiousness and mortality of ASF during the initial phase of an incursion is low to moderate while the case fatality rate is above 90%.

Therefore, ASF ought not be considered to be a highly contagious disease (21) and consequently, ASF control and eradication measures warrant a different approach to that of highly contagious diseases such as Foot and Mouth disease (FMD) or Classical Swine Fever (CSF) (Figure 2).

Nonetheless, many legislations worldwide have classified ASF as a highly contagious disease, as is the case in the Russian Federation (10), the Socialist Republic of Vietnam (12), the Commonwealth of Australia (9) and Canada (23), from the set of analyzed countries.

In the EU, legislation for CSF (a highly contagious disease) was employed as a template for ASF legislation: the CSF Directive (24) was used as a model for drafting the ASF Directive (25), following the same control and eradication measures for CSF.

If ASF is detected early and control measures are implemented without delay, the contagiousness has been demonstrated to be low (20). Virus spread within a farm as well as within a habitat is considered to be slow (7). The disease merely appears to be highly contagious in an environment where pigs are kept closely together and maintain frequent contact with other pigs within the pen.

During an outbreak investigation in 2017 on a large commercial pig farm (5,000 pigs) in Latvia, no deviation from the expected farm mortality rate was recorded during the first weeks of infection. More than 1 month passed before suspicion of ASF arose (26). This example demonstrates that under certain circumstances, i.e., in very large farms, early detection, within the first 2 weeks after virus introduction, can only be achieved through the regularly testing of sick and deceased animals (27). The presumptions made may slow down control and eradication and may even lead to further spread of the disease when a higher initial mortality rate is expected (“highly contagious disease”). Therefore, a surveillance scheme based on weekly sampling of deceased post-weaning pigs has been suggested as an early detection measure for ASF (28), particularly in holdings under risk of ASF incursions, e.g., where the virus is circulating in wild boar populations. In the current legislation of many countries, the characteristics of the disease such as low contagiousness/mortality and high case-fatality are not taken into account. Not specifying these characteristics may lead to the assumption that all disease scenarios are equal in this regard.

## Stamping out Measures

According to the current EU legislation (25), Member States shall ensure that in cases where ASF is officially confirmed in a holding, all pigs are to be killed without delay. However, exemptions to cull the entire farm can be made (25), based on a risk assessment and under a number of conditions that for the most part cannot be met in practice. The approach is similar in the USA (8).

The Republic of South Africa allows specific quarantine measures and not all animals must be culled within a holding (13). The Socialist Republic of Vietnam allows the slaughter of pigs for human consumption if these test negative for ASFV (12). Similarly, pre-emptive slaughter of healthy-looking pigs (often without testing) in an affected herd or area is practiced in a number of African countries where no compensation can be paid for culling and herd reduction is a preventive measure (14).

## Zoning

Recent events have highlighted that international trade can be significantly interrupted when ASF is detected even in just one region of a country, despite the fact that the principle of zoning is consistently applied, at least in the EU. However, since this concept is not always recognized by all trade partners, the entire pig sector of the country in question suffers the consequences of a ban on trade although only a few cases in a restricted area have been detected, even when they refer only to wild boar or non-commercial backyard pigs. Depending on the production system, economic consequences differ (29): zoning (the free area is defined by geography) may be the most cost-effective approach for small production systems, whereas compartmentalization (the free area is defined by husbandry practices related to biosecurity) may be the preferred approach for large commercial farms, due to the extensive areas covered (29).

Protection and surveillance zones in the EU need to measure a minimum of 3 km and 10 km respectively. The USA prescribes the same, including a buffer zone of 2 km between the two (8). In the Republic of South Africa “controlled areas” were established in 1935 in places where the sylvatic cycle is endemic (13). The Russian Federation establishes two distinct “threat zones,” the “first-threat” zone measuring a minimum of five to 20 km, the “second-threat” zone has a radius of 100–150 km (10). In the ASF-free Commonwealth of Australia, a 3 km “restricted area” will be established and the responsible authorities have flexibility regarding the control area which usually measures 10 km (9). China sets a 3 km radius in “infected areas” whereas the “threatened area” of 10 km will be extended to 50 km in areas of known wild boar activity. In all cases, a full epidemiological assessment must be conducted in order to estimate the extent of the outbreak (11).

## Surveillance

Surveillance activities in domestic pig populations are embedded within the various pieces of legislation relating to ASF. Variations exist regarding the locality of the surveillance measures applied and regarding the protocols and methods used for sampling and testing. For example, Canada pursues surveillance within and outside designated high-risk areas whilst Vietnam focuses its surveillance activities in its high-risk areas only (12, 23). EU legislation requires a minimum number of samples to be tested in the absence of clinical signs to detect 10% sero-prevalence with 95% confidence in infected areas (28, 30), whilst in the USA and Australia, the pattern and timing of testing may be determined according to the local disease situation and its specific circumstances (8, 9). In South Africa, apart from passive surveillance to identify outbreaks, active surveillance is based on monitoring of *Ornithodoros moubata* complex ticks from warthog burrows at the borders of the controlled area (31). Serological surveys are carried out in areas outside the controlled area after outbreaks to confirm absence of viral circulation (15).

In the European setting, disease surveillance in wild boar is carried out either by testing of all wild boar found sick or dead (passive surveillance is mainly aimed at the early detection of the virus in free at-risk areas), or by the testing of all hunted wild boar in an infected area, together with the testing of each dead animal (active plus passive surveillance). When virus prevalence and wild boar densities are low toward the end of disease eradication, the question whether active or passive surveillance is more efficient in detecting the virus is still open (32). EU legislation pursues both active and passive surveillance, whilst Canada's legislation predominantly focuses on passive surveillance as part of its control strategies in feral pigs (23).

## Compensation

Fair and timely compensation schemes ensure business viability and compliance with veterinary authorities. Farmers in the EU will receive compensation and the compensation modalities are organized in each Member State individually. The EU as the regional body provides the overall disease control framework and also contributes to compensation (33). In the USA and Australia (both currently ASF-free countries), only partial compensation is afforded with a cost-shared model operating between industry and government for the latter (8, 9). In China, compensation measures have become increasingly complex since 2018 when compensation for compulsory culling for ASF may have been at its highest. In February 2020, the Ministry of Agriculture and Rural Affairs of China (MARA) released the 2020 edition of the “ASF Epidemic Emergency Implementation Plan” in which it changed compensation measures depending on a number of factors, including cost-sharing arrangements between holdings where the outbreak occurred and the place of animal origin (11). Compensation for animals culled during outbreaks of controlled animal diseases was stipulated in earlier legislation in South Africa, but this has been rescinded for ASF (13). During outbreaks outside the control area, support was made available to subsistence farmers by industry and the Department of Social Development (15).

## Restocking

In the EU, restocking procedures are complex and restocking *per se* can only be permitted after a minimum of 40 days after cleaning and disinfection has been completed (25). In the USA, the local authorities can decide on restocking procedures depending on circumstances (8). Whilst in the Republic of South Africa no specific restocking procedures are laid down (13), in the Russian Federation, restocking can be undertaken within the “first-threat zone” only 1 year after quarantine removal (10). The legislation in China allows for restocking after a period of 5 months in addition to ASF-negative environmental samples; it also allows for restocking to take place 45 days after the introduction of sentinel pigs if these show no clinical abnormalities and produce negative test results (11).

The use of sentinel pigs as part of the restocking procedure varies. Sentinels are recommended to be used in the USA, South Africa, Canada only for outbreaks linked to ticks. The EU, Australia, Vietnam and Japan employ sentinel pigs regardless of ticks. Russia does not employ a sentinel system; the use of sentinel pigs in China is optional (8–10, 12, 13, 23, 25, 34). Sentinel pigs were used in several countries in West Africa before restocking (35), and served as core breeding stock in farms that did not receive compensation.

## Discussion

EU-legislation on ASF is about to change: from 21 April 2021, the so-called “Animal Health Law” will apply together with new regulations on disease listing, eradication programs, surveillance, prevention and control (36). Under the new legislation, ASF will be listed as a “Category A” disease and will continue to be subjected to rigorous prevention and control measures aimed at its eradication. However, compared to the current legal framework, there will be increased opportunities for each EU Member State to tailor ASF control measures, taking into account the local disease picture. Given the occurrence and persistence of ASF in several EU Member States, the Commission envisages safe trade and the smooth functioning of the EU single market through the implementation of the OIE-recognized principle of “zoning” *via* a new, specific Implementing Regulation (36).

Under this new legal framework, there will be opportunities to implement changes that improve outcomes and address specific problems posed by ASF in the EU. For instance, the most at-risk holdings vary according to the country, such as Estonia where commercial herds have been estimated to be more at risk than backyard farms (37), whereas the contrary has been reported from the Russian Federation (38).

African swine fever control measures in the EU largely follow the CSF control measures, based on the erroneous assumption that ASF is a highly contagious disease with a high mortality, affecting large numbers of pigs within a short time in an epidemiological unit, spreading readily from pig to pig and from farm to farm. However, analyses of domestic pig outbreaks in the current epizootic, as well as in experimental studies, revealed that the contagiousness is rather low and that under field conditions ASF virus transmission between animals can be slow (20). The principle aim is to eradicate the virus within affected zones and allow for the trade of animals and animal products outside the restricted areas in order not to disrupt commerce.

Japan and the USA do not define ASF to be a highly contagious disease (8, 34) and relevant legislation characterizes ASF as “a typical example of a transboundary animal disease” defined by international organizations such as the FAO as “a disease that spreads across national borders and is of importance to the economy, trade, and food security of the outbreak country and requires multilateral cooperation to prevent its epidemic” (34). South African legislation does not mention contagiousness, only describing the different transmission routes (13).

Anthropogenic activities have been identified as the main drivers for disease transmission in the domestic pig cycle and are responsible for long-distance jumps of disease in wild boar (20, 21), as opposed to animal-to-animal transmission, which has also been recently described for domestic pigs in South Africa (14).

The sound implementation of any early detection surveillance scheme will enable the detection of potentially infected holdings in the early stages of disease progression with only few virus-positive animals present. While early detection and removal of infected animals is crucial to eliminate or reduce the risk of virus transmission, on-farm depopulation or preventive culling often lead to highly emotional and difficult situations where farmers refuse to accept depopulation measures when they do not see the justification for drastic measures.

Environmental complications arise when a high number of carcasses are disposed of *via* incineration or burial. The benefits of effective disease control must be balanced against costs and ethical consideration of the control measures applied. Excessive culling raises ethical issues when more pigs are culled than deemed necessary to prevent disease spread. In the Netherlands in 1997, ~11 million pigs were culled to combat CSF whereas <1 million were actually infected (39).

Nevertheless, the stamping-out policy seems entirely justified where it leads to rapid disease eradication and a return to normal trade, i.e., where intensive, trade-oriented pig farming is an important economic activity. Conversely, it seems questionable under other contexts, where this policy does not effect clear advantages, either in epidemiological or in economic and social terms and is at odds with safeguarding animal welfare. This is the case when ASF cannot be swiftly eradicated due to biological reservoirs other than domestic pigs and where backyard/non-commercial pig farming prevails. Under these circumstances, alternatives to a stamping out policy should be explored and reflected in legislation.

If good on-farm surveillance can be established *via* the use of modern diagnostic techniques (e.g., sensitive and specific pen-side tests) the number of animals destined to be destroyed on an affected farm could be reduced. Equally, targeted culling programs of infected contact animals could be employed, based on veterinary risk assessments that take into account the characteristics of the biological agent, the farming system, biosecurity and distances between animal groups (40).

Zoning is one of the early actions to be employed in case of an ASF incursion into a country. Many countries request 3 km and 10 km zones around outbreak points (e.g., EU, Australia, Japan). Those radii largely remain a proven tool for controlling and eradicating highly contagious diseases such as CSF, although in very densely populated areas preventive culling may need to be applied as an additional measure. Relying on the epidemiological results enables the local authorities to choose radii that are scaled to the threat. In the case of ASF control and prevention, efficient epidemiological tracing of potentially infected farms may replace the zoning strategy, avoiding the implementation of zones over 3 km radius. On the other hand, larger zones may be chosen for ongoing infections in wild boar populations. The proximity of wild boar to both backyard and commercial farms is a risk factor in the emergence of ASF outbreaks in domestic pigs, which is even more impactful when the level of biosecurity is low or when wild boar abundance is comparatively significant (41). According to the local context, the increase in ASF cases in wild boar can even be the main risk factor leading to outbreaks in pig farms (37).

Ideally, tracing activity and compartmentalization should supplement any zoning strategy. The concept of compartment widens the geographical approach of zoning by going beyond the “risk borderline.” It incorporates all epidemiological elements that allow to define more appropriately an effective boundary and should ideally be defined before an outbreak occurs (42). However, to maintain international pork trade for countries facing cases of ASF in wild boar or domestic pigs, a binding international agreement is required on how the safety of pork products can be guaranteed (43).

As zoning may restrict animal movements and trade potentially more than necessary regarding high-biosecurity holdings, implementing compartmentalization for eligible pig units could be viewed as a compromise between business disruption and disease control. An issue that is still discussed by the EU working group on zoning and compartmentalization is the possible scenario of a compartment being located close to a disease outbreak, for example in the surveillance or protection zone. This scenario has not yet been sufficiently considered at an international level (44). Until a derogation is issued, intra-community transport in relation to the compartment will not be permitted under existing EU legislation. An early and short standstill would apply, in order to ensure that the compartment's integrity is maintained. The EU working group is currently developing procedures to improve the management of this scheme (44).

If, for example, only backyard farms are affected, the size of restricted zones could be rapidly reduced (or derogations could be made to allow animal movements) once it has been established that commercial farms within the zones are not involved; the impact on trade would be reduced. In the current situation, commercial enterprises are keen to see that outbreaks in backyard farms are dealt with rigorously and without delay in order to avoid long lasting restrictions themselves.

The ideal radius could be determined based on local farm density and the levels of biosecurity. Infection probabilities of neighboring premises can be ascertained for the main TADs and could be readily applied if the geographical location of each farm was established; in this case, the radius would be defined according to the local conditions under a specific strategy set at national/international level; without it, the 3 km radius remains an accepted simplification.

Economic consequences differ according to the production system. It has been estimated that zoning (the free area is defined by geography) would be the most cost-effective approach for small production systems, whereas compartmentalization (the free area is defined by husbandry practices related to biosecurity) would be better for large commercial farms, due to the extensive areas covered (29). The latter study focused mainly on live pig trade though, whereas the movements of live pigs is not the only transmission route for ASF.

Considering the numerous disease-specific interdependencies and the potential means of transmission (e.g., fomites), many ASF action plans have been tested throughout the world in the last decades. A study from 2016 (45) compared twenty surveillance strategies regarding ASF mitigation. The study highlighted the importance of disease-specific intervention strategies that need to be effective and practical. It concluded that the best surveillance strategies include pig mortality assessments at farm-level [defined as the use of observable mortality-related data before confirmed diagnoses are made (46)] and carcass assessment in relation to wild boar.

The contribution of wild boar regarding disease spread is widely accepted and acknowledged in various pieces of legislation worldwide. “Wild boar are a significant risk factor for disease transmission in general […]. The presence of wild pigs is the most predictive risk estimate of disease spread” (23). In wild boar populations, ASFV can survive in the local population with a low prevalence below 5% and a transmission speed of 2–5 km/month (20). The low contagiousness of the virus is compensated for by its high tenacity (i.e., pork products, environment, etc.). Carcasses of ASF deceased wild boar allow the virus to persist for months or even years in a given area. It is estimated that the persistence of the virus in carcasses, and its spread through carcasses, is more important than direct contact with live infectious animals (23) when at low population density.

The main strategic aims of surveillance in domestic pigs are the early detection of potentially infected holdings and proof of freedom from the disease in a region/country after a disease event in order to lift restrictions. Surveillance is compulsory within protection and surveillance zones around outbreak holdings as well as in holdings located in areas that are under restrictions due to the presence of ASF in wild boar.

Nowadays, effective surveillance is mainly based on passive surveillance, targeting sick and deceased animals that are to be tested for the presence of ASF virus. The passive surveillance approach is based on the fact that ASF case fatality is often very high (>90%), signifying that almost all animals that pick up infection will become sick and die. The low contagiousness of ASF results in only few animals affected at the beginning of an infection in a given holding (21). Seropositive animals can be identified only during an advanced stage of an epidemic (28). As there is a very short time from infection to death [3–10 days (20)] and as the case-fatality rate is close to 100%, surviving, and thus ASF-seropositive animals, can hardly be found. Therefore, active surveillance based on random serological testing is no longer recommended in regards to the early detection of ASF. The EU diagnostic manual for ASF (which took into account the experience in the Iberian Peninsula and in Sardinia, where seropositive animals were very common) (30) still prescribes random blood sampling to determine antibody-positive domestic pigs. However, as shown in a recent EFSA report (28), serological surveillance would still not lead to early detection of disease.

ASF- legislation from Japan summarizes as follows: “African swine fever has a short course from infection to death, and most cases do not show elevated antibody titers, making serologic tests less useful as a diagnosis. For rapid diagnosis, genetic testing such as conventional PCR, which specifically detects the ASFV gene, is the most effective” (34). In terms of surveillance, the lengthy persistence of antibodies means that these can be found long after any viable virus has disappeared. In the absence of recent outbreaks, detected antibodies must not be understood to be a proof of “silent circulation” of the virus, although antibodies can be a valuable tool in endemic areas in particular toward the end of an epidemic; screening for antibodies can be a valuable tool for lifting restrictions.

Equally, in areas which are under restriction due to ASF in wild boar, it is recommended to conduct passive surveillance in domestic pig holdings and to sample a number of specified animals in each production unit (27).

The role of pigs surviving the disease continues to be controversially discussed. However, old (47) and new (48, 49) studies could not demonstrate that animals that survive the disease play a significant role in disease spread (50). Despite this fact, EU legislation does not differentiate between exclusively seropositive and PCR positive cases. For outbreaks in domestic pig farms, this is not relevant as the entire herd is culled after confirmation of disease but for the management of ASF in wild boar this remains a major concern. In some areas that experienced ASF during the past 5–6 years, all reported cases were limited to sero-positive, healthy hunted wild boar; such areas are therefore struggling with, one could argue, unjustified consequences such as trade restrictions (51).

ASF in mainland Europe is gradually changing: while serological tests are of limited importance in areas that have been recently affected, serology becomes an important additional tool that allows us to better understand how the disease spreads, and evolves, in areas where the virus is circulating or has been circulating for a long time. In Sardinia, serology remains a very important tool (6).

Many countries include compensation in the framework of their respective disease control measures (e.g., China, Russia). Compensation schemes vary, from full compensation (e.g., Japan, EU) to partial compensation (e.g., the USA). At best, adequate compensation payments will incentivize farmers to report suspicion of disease and may generally aid in matters of compliance relating to on-farm disease interventions by the responsible authorities. At worst, with little or no compensation, suspicion of disease may not be reported to the responsible authorities and instead farmers choose to hastily slaughter or sell their sick pigs at local markets, or dispose of carcasses illegally. Such circumstances have been recognized as a major cause of disease spread (35, 52, 53).

Conversely, overcompensation may lead to a situation where a farmer who expects to receive compensation in the event of a disease outbreak has weaker incentives to avoid risk during “peace times.” This issue can be prevented if payments are made on the condition that farmers adhere to specific biosecurity practices (54). As far as possible, compensation schemes should be carefully reviewed and improved where necessary. Innovative compensation schemes could potentially reduce the costs of control measures due to early mitigation of an outbreak. Replacement of (core) breeding stock in lieu of direct financial compensation could be considered, especially for small farming enterprises.

Following the lifting of restrictions, the time after which restocking can be attempted, varies considerably according to the relevant legislation across the globe. It can range from 1 month in the case of Vietnam to 1 year in the case of Russia, or even 6 years for the EU if the outbreak has been linked to ticks. It is unrealistic to assume that a farming enterprise will be able to hold out financially for years until restocking can take place, hence in practice this is neither affordable nor a realistic approach.

Regarding disease eradication measures, progressive legislation will take into account farming practices at the opposite ends of the spectrum, namely commercial farm enterprises and backyard farm systems. Biosecurity measures that warrant compliance were based on modern farm enterprises and cannot be readily transferred onto, or realized on, traditional backyard settings. Measures imposed on backyard farms that cannot be realized due to cost or the given farm infrastructure, may lead to compliance fatigue; farmers may abandon traditional farming practices altogether, potentially leading to the loss of rare breeds and the loss of cultural identities of many nations.

When dealing with small non-commercial producers the EU Directive for the control of avian influenza, for example, considers different measures to those employed on large commercial enterprises (55). Although highly pathogenic avian influenza is considered highly contagious (as opposed to ASF), there are a number of derogations for non-commercial holdings where animals are kept either as pets or for own consumption. Derogations exist also for culling, establishment of protection and surveillance zones, visits by the official veterinarian and surveillance. Such derogations aid the official veterinarian when dealing with non-commercial holdings and could be adapted to the situation of ASF in the backyard sector.

ASFV is a very complex virus and our understanding continues to evolve in parallel with its current, unprecedented spread. We are not yet in a situation to draw conclusions on a single, “worldwide valid” disease control strategy and the legislation that requires its control and eradication. Strategies based on farming systems would provide the flexibility that a global and rigid disease control strategy cannot offer.

In Sardinia, an ASF scenario emerged that largely differed from the one in mainland Europe: free ranging pigs represented the main ASF reservoir whilst infection in wild boar played merely an ancillary role (6, 56). This specific disease scenario may be in large part due to the long evolution of ASFV, over four decades, where a large proportion of affected animals (at least free-ranging pigs and wild boar) survive the disease (6). Accordingly, a disease control strategy was implemented by the local authorities in recent years that targeted illegally kept free ranging pigs (where virus prevalence was never higher than 2–3%, while sero-prevalence reached 70%). This led also to a major, rapid drop of virus circulation in wild boar and confirms that in the Sardinian scenario wild boar merely play(ed) an ancillary role in disease transmission.

## Conclusions

- Based on the spirit of the new EU Animal Health Law, future legislation should take into consideration the disease-relevant characteristics of a biological agent, biology of disease and its epidemiological profile as well as specific pig husbandry traditions. Animal Health related legislation, policies and strategies should be revised in cases where gained scientific knowledge can improve disease control, leading to continued evidence-based policymaking.
- Detailed epidemiological farm investigations, combined with a surveillance scheme based on enhanced passive surveillance must be implemented. Epidemiological tracing of contact farms is of paramount importance in order to identify sources of infection as early as possible and to interrupt the spread of the disease.
- Holdings located outside a restricted area, but linked through human activity to an infected farm, can constitute a higher risk than holdings that implement good biosecurity within a protection or surveillance zone. In this context, a review of the size of restriction zones and studies that evaluate the effectiveness of a given surveillance zone (i.e., 10 km) in relation to the prevention of ASF could be of value.
- Taking into account the relatively low contagiousness of ASF and its relatively slow spread, smaller zones (<3 km) could be considered for outbreaks in domestic commercial pig holdings whilst focusing efforts on epidemiological tracing to detect potential contact farms.
- Effective surveillance for early detection of ASF infection should focus on virus detection and differentiate between exclusively seropositive and virus positive animals especially in the wild boar context.
- Alternative culling schemes for large farms, at which only few infected animals have been detected at an early stage, should be developed. Good managerial and strict internal biosecurity measures as well as intelligent farm surveillance schemes would pave the way for reaching this goal. Early detection remains a key priority.
- When dealing with non-commercial holdings, derogations for smallholders should be considered in order not to put traditional self-sustaining agriculture at a disadvantage and to ensure survival of these traditional farming methods that express the cultural identity of many countries—and that contribute to conserving genetic resources through the keeping of rare and traditional breeds.
- Global trade could suffer fewer interruptions if legislation, in line with OIE standards, considered the zoning principle; this would not prohibit all imports from the whole of a country but only from its well-defined infected areas, in case of a localized outbreak.

## Author Contributions

FB and KDe conceptualized and designed the overall study. CH and FB collected and analyzed the data. FB drafted the manuscript. CH, M-LP, AL, KDi, AG, VG and LZ edited the manuscript. All authors contributed to the article and approved the submitted version.

## Conflict of Interest

The authors declare that the research was conducted in the absence of any commercial or financial relationships that could be construed as a potential conflict of interest. The reviewer KS declared a past co-authorship with the authors M-LP, KD, VG to the handling editor.
